# Investigation of the Reinforcement Mechanism and Impact Resistance of Carbon Hollow Microsphere-Reinforced PDMS Composites

**DOI:** 10.3390/polym17152087

**Published:** 2025-07-30

**Authors:** Yingying Yu, Yaxi Zhang, Cheng Yang, Fandong Meng, Fanyi Meng, Tao Wang, Zhenmin Luo

**Affiliations:** College of Safety Science and Engineering, Xi’an University of Science and Technology, Xi’an 710054, China

**Keywords:** carbon hollow microsphere, polydimethylsiloxane, mechanical properties, energy absorption, strain rate effect

## Abstract

For meeting the growing demand for lightweight impact-resistant materials, this study designed and fabricated a carbon hollow microsphere (CHM)-reinforced polydimethylsiloxane (PDMS) composite and systematically investigated the influence of CHM packing structure on its energy absorption performance. Through optimizing the controllable preparation processes of the CHMs, CHMs with low breaking rates and novel structural stability were successfully prepared. A vacuum-assisted mixing–casting method was employed to synthesize the CHM/PDMS composites with varying CHM contents (0~10 wt.%). The results demonstrated that the incorporation of CHMs significantly enhanced the compressive strength, compressive modulus, and energy absorption efficiency of the PDMS matrix. Under quasi-static loading, the composite with 4 wt.% CHM exhibited optimal comprehensive performance, achieving a 124.68% increase in compressive strength compared to pure PDMS. In dynamic impact tests, the compressive strength and energy absorption at a strain rate of 4500 s^−1^ increased by 1245.09% and 1218.32%, respectively. The improvement of mechanical properties can be mainly attributed to the introduction of CHMs with an appropriate percentage, which can form a dense stacking structure so that the interaction force between the CHMs and PDMS matrix can be improved through the dense stacking effect, and the external force can be effectively dissipated through interface interaction, in addition to the energy dissipated by the deformation of the matrix deformation and crush of the CHMs. Additionally, the introduction of CHMs elevated the onset thermal decomposition temperature of the materials, leading to an enhanced thermal stability of the CHM/PDMS composite compared to that of the pure PDMS. Overall, this study provides theoretical and experimental foundations for designing lightweight impact-resistant materials and demonstrates the potential of CHM/PDMS composites for multifunctional safety protection.

## 1. Introduction

The chemical industry is developing rapidly. However, its high safety risk has led to an increasing demand for protective equipment with excellent impact resistance. Although traditional metal materials exhibit outstanding mechanical strength, their high density restricts their application in lightweight equipment. Consequently, porous materials with low density, high specific strength, and superior impact resistance are gradually emerging as new structural and functional materials for light-weight energy absorption equipment. Polysiloxanes are a class of polymeric materials composed of a silicon–oxygen backbone, with structures formed by O–Si–O and Si–O–Si bonds, showing excellent molecular chain flexibility [1]. Among these polysiloxanes, polydimethylsiloxane (PDMS) is one of the most extensively studied and high-performing siloxane polymers [2]. Owing to its excellent damping capacity, low glass transition temperature, and environmental friendliness, PDMS is widely applied in wearable devices, smart electronics, and light-emitting diodes (LEDs) [3,4,5,6]. However, pure PDMS elastomers typically exhibit relatively low mechanical strength, which limits their application in the scenarios that require high mechanical strength [7].

Thus, research was carried out to optimize the mechanical strength of PDMS by introducing high-strength nanofillers [8]. Previous studies have shown that, without significantly increasing material weight or compromising processability, a moderate amount of filler can effectively enhance the mechanical performance of the polymer matrix [9,10,11]. In particular, the induction of hollow microspheres can construct low-density porous composites, with the overall density adjustable by controlling the hollow microsphere content. Such composite foams exhibited excellent mechanical properties, enabling them to absorb large amounts of energy under compressive loads [12]. Compared to other closed-cell structures, these composite foams offered higher strength, excellent impact resistance, and good damage tolerance. For example, Zhao et al. [13] incorporated fly ash cenospheres (FACs) into a PDMS matrix to fabricate a rigid–soft structured composite material. This composite not only had a low density ($80.668\;g/{\mathrm{cm}}^{3}$) but also exhibited a low thermal conductivity (0.39 W/mK) and excellent specific energy absorption (8.13 J/g at $2.8\;\times {\;10}^{-3}\;s^{-1}$ and 15.49 J/g at $3000{\;s}^{-1}$). In addition, Yang et al. [14] produced a structurally stable PDMS/graphene oxide (GO) aerogel (PGOA) by in situ adhering PDMS chains onto GO nanosheets, which was subsequently reduced to PDMS/graphene aerogel (PGA). The as-prepared PGA exhibited a compressive strength of 1.7 MPa and low thermal conductivity (0.0245~0.0301 W/(m·K)). In addition, PGA also owned excellent flame retardancy with heat resistance up to approximately 1000 °C.

The geometry of carbon fillers significantly affects the mechanical performance of the materials [15]. The reinforcing effect should be governed by the morphology of the carbon materials and the interfacial interactions, which determine their distribution within the matrix and the degree of reinforcement. Since the discoveries of fullerenes, carbon nanotubes, and graphene, various forms of carbon materials have exhibited different dispersibility and orientation in polymers, due to their unique geometrical features, thereby influencing the composite’s strength, toughness, and stiffness [16,17,18]. For instance, carbon nanotubes can markedly enhance the flexural strength and toughness of composites, whereas spherical carbon materials promote uniform filler dispersion [19]. As a new member of the carbon material family, carbon hollow microspheres (CHMs) have attracted widespread attention over the past decade due to their high specific surface area, low density, excellent thermal stability, good electrical conductivity, chemical inertness, and superior compatibility with matrices [20,21,22,23,24]. For example, Zheng et al. [25] synthesized monodisperse mesoporous carbon spheres (MCSs) and compounded them with ethylene propylene diene monomer (EPDM) rubber on a two-roll mill to form MCS/EPDM rubber composites. The results indicated that the addition of MCSs significantly improved both the tensile strength and elongation at break of the EPDM foam, as EPDM molecular chains penetrated the pores of the MCSs and thereby enhanced the interfacial interaction between the filler and the matrix. Phenolic resin, a polymer formed by the polycondensation of phenol and aldehydes, is widely used due to its simple synthesis process, high carbon content, excellent thermal stability, flame retardancy, radiation resistance, and friction properties. Thus, it is an important precursor for synthesizing carbon spheres [26]. However, studies on the mechanical properties of soft elastomer materials reinforced with CHMs under both low and high strain rates remain relatively limited.

To address this issue, the present study designed and synthesized a lightweight composite material based on CHM/PDMS. The focus was on investigating the effect of the CHM filler packing structure on the composite’s mechanical properties, with a systematic evaluation of its compressive strength and energy absorption capacity under quasi-static and dynamic loading conditions. Furthermore, a dynamic impact factor (DIF) model was established to characterize the mechanical response of CHM/PDMS composites under high strain rates, complemented by scanning electron microscopy (SEM) analysis of the micro-damage mechanisms. The findings not only provide theoretical and experimental support for the application of CHM/PDMS in impact-resistant engineering but also demonstrate the potential value of the composite in various engineering fields.

## 2. Experiment

### 2.1. Preparation of the CHMs

**Figure 1:**
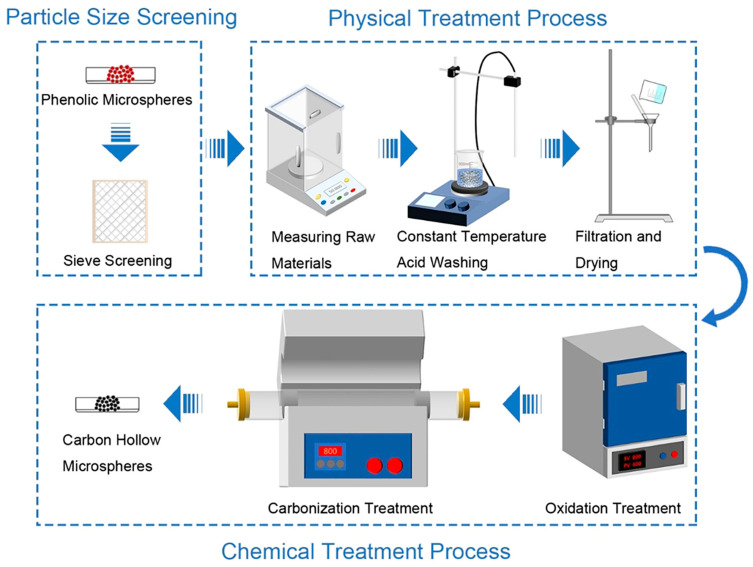
The schematic illustration of the four-step process for CHM preparation.

The PDMS matrix (BD6184) was purchased from Hangzhou Guinee Advanced Materials Co., Ltd. (Hangzhou, China). The precursor of the CHMs, the phenolic microspheres (BJO-0930), were supplied by Maroya New Material (Shanghai) Co., Ltd. (Shanghai, China). The average density and the compressive strength of the phenolic microspheres were 0.104 g/cm^3^ and 3 psi, respectively. The CHMs were synthesized through a four-step process as illustrated in Figure 1. Initially, the phenolic microspheres with non-uniform sizes were sieved by multi-size sieve mesh for improving the size uniformity and avoiding uneven stress caused by the non-uniform microsphere size. Metal elements can be found on the surface of the phenolic microsphere, as shown in Figure S1. It can be inferred that these metal elements exist on the surface of the microsphere in the form of metal oxides. Metal oxides can cause the microspheres to be heated unevenly during the carbonization process, which is not desirable for improving the structural stability of CHMs. As a result, the phenolic microspheres were added to a 0.1 mol/L hydrochloric acid solution to remove the metallic impurity present on their surfaces. After the acid treatment, the phenolic microspheres underwent a pre-oxidation process at a temperature of ~180 °C for 12 h, which cross-linked the phenolic hydroxyl groups on the microsphere surface into ether bonds, improving the toughness and thermal stability of the microspheres. Finally, the oxidized microspheres were carbonized at the temperature of 800 °C for 2 h, with a heating rate of 1 °C/min. Through this four-step process, the CHMs with controllable size were prepared. The as-prepared CHMs with varying diameter distribution of microspheres are depicted in Figure S2.

### 2.2. Preparation of the CHM/PDMS Composites

To investigate the influence of CHM content on the energy absorption performance of the CHM/PDMS composites, a series of CHM/PDMS composites with varying CHM contents (2 wt.%, 4 wt.%, 6 wt.%, 8 wt.%, and 10 wt.%) were synthesized via a vacuum-assisted mixing–casting process. For reference, pure PDMS samples were also prepared.

The PDMS material consists of two basic components: a prepolymer and a curing agent. The main component of the prepolymer is low molecular weight PDMS terminated with vinyl groups. The curing agent is composed of hydrogen-terminated PDMS and includes a catalytic element, specifically a platinum-based compound. The PDMS was prepared with a weight ratio of 10:1 (base to curing agent). The preparation of the CHM/PDMS composites followed a simple two-step process of mixing and casting. First, a predetermined amount of PDMS was placed in a beaker, followed by the addition of the desired weight fraction of filler (i.e., CHMs). To prevent agglomeration, the CHMs were added to the matrix in multiple steps, between which the mixture was gently stirred to minimize air bubbles and obtain a uniform precursor. Then, the curing agent was added to the well-mixed precursor. The sample underwent a two-hour degassing process to ensure complete bubble removal. The mixture was then poured into a polytetrafluoroethylene (PTFE) mold sprayed with a silicone-based release agent to form samples of standard dimensions. The curing process involved an initial cure at 60 °C for 0.5 h, followed by post-curing at 100 °C for 2 h.

The as-prepared CHM/PDMS composites were denoted by CHM*_x_*/PDMS, where *x* represents the mass fraction of the CHMs in the composites. For example, CHM_02_/PDMS denotes the composite containing 2 wt.% CHMs.

### 2.3. Characterization of CHMs and CHM/PDMS Composites

The quasi-static compressive performance of the as-prepared materials was obtained through an Instron 3365 universal testing system with a 5 kN load cell. Cylindrical specimens with a diameter of 10 mm and a height of 7 mm were designed, and the crosshead displacement speed was set to 1 mm/min. For each group, three replicate tests were conducted under displacement-controlled mode. Dynamic impact tests were performed using a split Hopkinson pressure bar (SHPB) system. The voltage signals were obtained through the SHPB system and converted into stress–strain curves based on the one-dimensional stress wave theory. Dynamic impact tests were carried out at strain rates of approximately 2500 s^−1^, 3500 s^−1^, and 4500 s^−1^, with each group undergoing at least five repeated tests. Cylindrical specimens with a diameter of 6 mm and a height of 2 mm were prepared for dynamic impact tests. The microstructure of the samples was measured using a scanning electron microscope (Quanta 450, FEI, Hillsborough, OR, USA) equipped for energy-dispersive X-ray spectroscopy (EDS, IE 250X-Max 50, Oxford Instrument, Abingdon, UK). The thermal degradation behavior was examined with TG (TG 209 F1, Netzsch, Gebrüder, Germany) under a nitrogen atmosphere with a heating rate of 10 °C/min from 25 °C to 900 °C.

## 3. Results and Discussion

### 3.1. Quasi-Static Compressive Performance of the CHM/PDMS Composite

**Figure 2:**
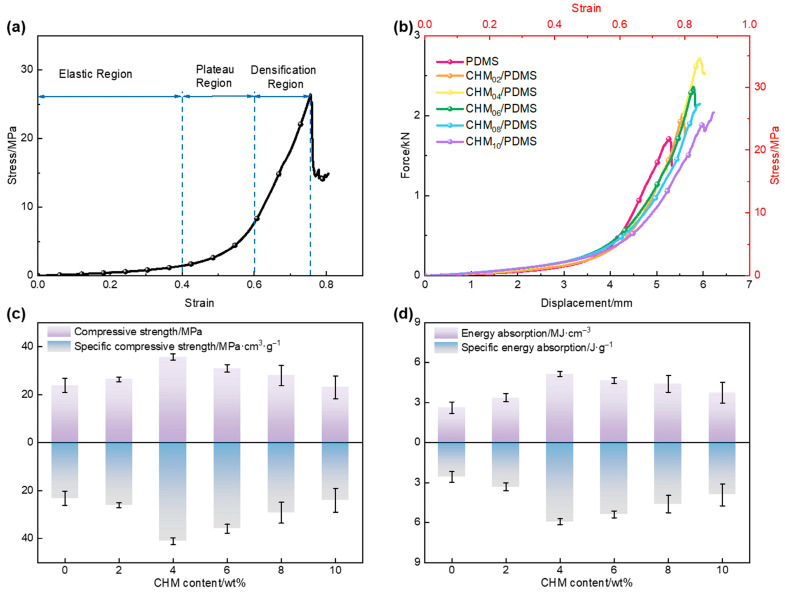
Quasi-static mechanical properties of CHM/PDMS composites: (**a**) typical quasi-static stress–strain behavior curve; (**b**) stress–strain curves and force–displacement curves; (**c**) compressive strength and specific compressive strength; (**d**) energy absorption per unit area and energy absorption density.

A representative quasi-static stress–strain curve of each CHM/PDMS composite is displayed in Figure 2a. The curve can be divided into three regions: elastic region, plateau region, and densification region. In the elastic region, the specimens with uniform CHM distribution undergo uniform deformation, giving rise to a linear increase. With the load increasing, the stress shows an accelerated increase due to part of the CHMs rupturing, forming a plateau region. Before the stress reaches its peak, a yield point is supposed to be observed during the elastic region, followed by a plateau region with higher stress increase rate compared to the elastic region, which is the typical characteristic of soft polymer materials [27]. The energy absorption of the CHM/PDMS composite under the low strain rate can be partly related to the rupture of the microspheres, which exposed their hollow interiors to accommodate the compressed material [28]. When the number of crushed microspheres exceeds a critical value during compression, the stress begins to rise steeply, marking the densification region. Then, the stress reaches its maximum value followed by a dramatic drop, indicating the onset of crack initiation in the matrix. Figure 2b presents the stress–strain and force–displacement curves of composites with varying CHM contents. Combined with the results shown in Figure 2c,d, it is evident that, in the 0 wt.%~4 wt.% CHM content range, the compressive strength of the composites increased proportionally with enhanced CHM content, reaching the highest value of 35.8 MPa. This is due to the significant difference in elastic modulus between the CHM and PDMS: CHM exhibits an elastic modulus of several tens of gigapascals, which is far higher than that of the PDMS matrix. Moreover, the result indicates that CHMs worked as the reinforcement component, which primarily bore the compressive load within the composite, far exceeding the load on the PDMS matrix. Incorporating such a high-modulus filler into the matrix can significantly enhance the overall stiffness of the composite.

However, as the CHM content increases from 4 wt.% to 10 wt.%, the compressive strength of the composite becomes inversely proportional to the CHM content, indicating that increased filler content leads to poorer dispersion and agglomeration within the matrix, potentially resulting in incomplete curing of the matrix [29]. Throughout the different stages of the stress–strain curves, noticeable differences in the slopes were observed. Notably, at higher strains, the slope of the pure PDMS matrix curve gradually exceeds that of the composite, suggesting that the composite undergoes progressive damage during loading, which is different from the pure PDMS. The energy absorption performance of the materials showed a similar trend with their compressive strength, and CHM_04_/PDMS samples were demonstrated to have the best energy absorption performance of 5.1 MJ/cm^3^, which was 109% higher than that of the pure PDMS.

**Figure 3:**
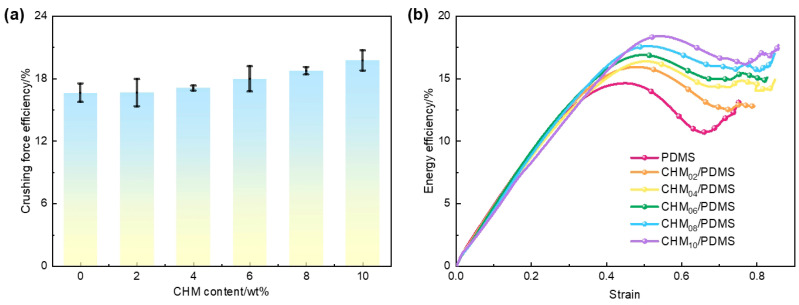
Mechanical performance evaluation of CHM/PDMS composites: (**a**) crush efficiency; (**b**) energy absorption efficiency curves.

Energy absorption efficiency curves reflect both the elastic and plastic behaviors of materials. Figure 3 illustrates the variation in crush efficiency (CFE) and energy absorption efficiency (*η*) of the CHM/PDMS composites with varied CHM contents under quasi-static compression. Unlike compressive strength and energy absorption values, both CFE and *η* increase as the CHM content rises, indicating a differentiation in material performance across different mechanical response stages. This phenomenon occurs because, when the CHM content exceeds 4 wt.%, the dispersion of fillers in the PDMS matrix progressively deteriorates, leading to localized agglomeration. The agglomerations act as stress concentration points, triggering interfacial debonding or microcrack propagation, which results in local failure at lower strains and a reduction in compressive strength.

On the other hand, as the CHM content increases, a larger proportion of the matrix can be occupied by the CHMs, restricting the viscoelastic flow of the PDMS. The stress concentration could not be relieved easily through plastic deformation, which is not desirable for improving the load-bearing and energy absorption performances. With higher CHM concentration, more hollow microspheres participate in the compression process. Although part of the microspheres can be ruptured, the intact ones continued to absorb energy through plastic deformation. Based on the force conduction, the plastic deformation of the microspheres continuously occurs, which can lower the peak load while maintaining a high average crush force, thereby enhancing the crush efficiency. Additionally, a high CHM content extended the strain range through microsphere deformation or rupture, allowing for more sustained energy absorption. Consequently, the decrease in energy absorption per unit area was less pronounced than the reduction in the peak compressive stress, leading to an increase in *η*.

**Figure 4:**
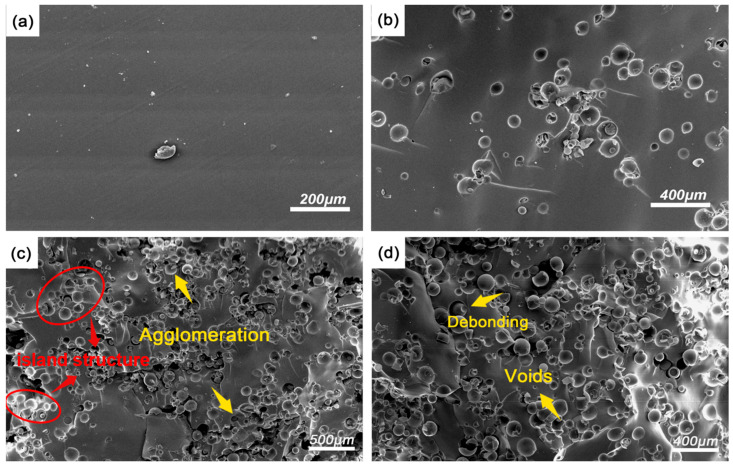
The SEM images of the materials under quasi-static loading. (**a**) PDMS; (**b**) CHM_02_/PDMS; (**c**) CHM_06_/PDMS; (**d**) CHM_08_/PDMS.

Figure 4 shows the SEM images of the fracture surfaces of CHM/PDMS composites under quasi-static compression. Figure 4a displays the SEM image of pure PDMS under quasi-static loading, where the micro-surface is mostly smooth with only a limited number of depressions scattered across the fracture surface, indicating that pure PDMS possesses a certain degree of ductility. For pure PDMS, the number of the microcracks within the matrix is relatively low and can be mainly attributed to the lack of crack branching and deflection. It can be seen that the CHMs are uniformly dispersed within the PDMS matrix with no obvious agglomeration when the CHM content is 2 wt.% (Figure 4b). In addition, the edges of the microspheres show no signs of debonding, and the CHMs are tightly bonded with the PDMS matrix, forming a “rigid microsphere–soft matrix” composite structure. When the CHM content reaches 6 wt.%, the dispersion of the microspheres deteriorates, which increases local agglomeration and reduces inter-microsphere spacing in some regions, though a certain degree of continuity can be still maintained.

However, when the CHM content reaches 8 wt.%, severe agglomeration leads to the formation of “island structure” (as indicated by red circles and arrows in Figure 4c), where agglomerated microspheres act as the island, resulting in the appearance of obvious pores and cracks accompanied by interfacial debonding [13]. At this point, due to the modulus mismatch between the PDMS matrix and the CHMs, significant permanent debonding occurs among the fillers and the matrix, and noticeable voids can be found where the filler is embedded, indicating that interfacial debonding could be the primary mode of damage induced by compressive stress. The failure mechanism of the CHM/PDMS composites can be concluded as follows: As CHMs are incorporated in the PDMS matrix, the rigid microspheres act as the barriers to hinder the crack propagation, which significantly alters the crack paths. When a propagating crack encounters the CHMs, it cannot directly pass through the rigid microspheres and tends to detour to continue its propagation. However, when microcracks from different planes intersect, local stress transfer through the matrix can be weakened, leading to a sharp increase in stress concentration at the crack interface.

### 3.2. Dynamic Compressive Performance of the CHM/PDMS Composite

**Figure 5:**
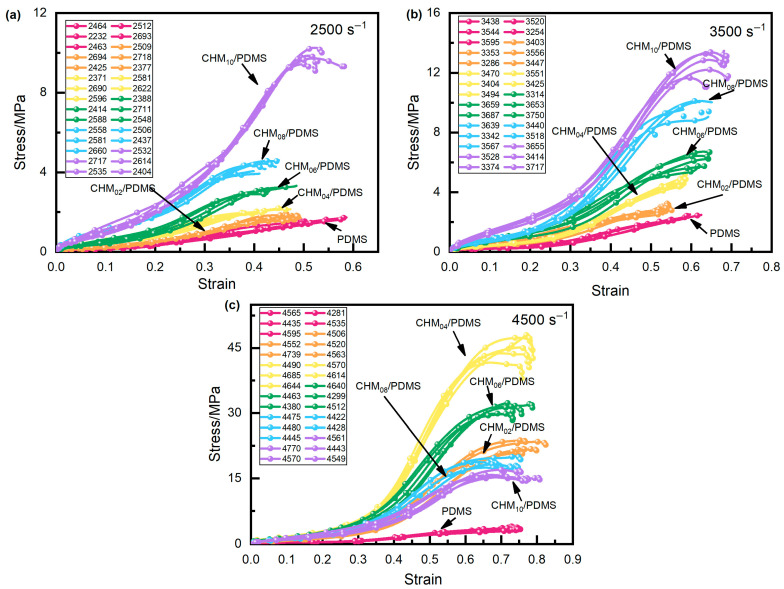
Stress–strain curves of the materials under dynamic impact at strain rates of (**a**) 2500 s^−1^; (**b**) 3500 s^−1^; and (**c**) 4500 s^−1^.

The strain rate induced by the SHPB system is controlled by adjusting the air pressure of the gas gun, and the strain rate at high strain rates is not constant but varied within a certain range. In this section, the engineered stress–strain performances of CHM/PDMS composites under high strain rate (approximately 2500 s^−1^, 3500 s^−1^, and 4500 s^−1^) were carried out, as shown in Figure 5. Each sample with a different CHM content was measured five times and the exact strain rates are shown in the legend. The color of the curves is used to distinguish the samples with different CHM contents. Compared to the pure PDMS samples, the incorporation of CHMs enhances the impact resistance of the materials, which can be attributed to the interaction between the CHM fillers and the PDMS matrix. The interaction prolongs the time required for the composite to fracture under impact loading and increases its impact strength, mitigating failure under such conditions. Furthermore, at lower strain rates (2500~3500 s^−1^), the stress in the CHM/PDMS composites gradually increases with strain, exhibiting typical nonlinear elastic behavior and strain rate hardening characteristics. At a higher strain rate (4500 s^−1^), the increase rate of the stress shows a trend of initially rising, then falling, and rising again, which can be attributed to changes in the dynamic response of the CHM/PDMS composites at high strain rates. Moreover, when examining composites with the same filler content, it can be observed that at a given strain, the stiffness at a strain rate of 4500 s^−1^ is noticeably higher than that at strain rates of 3500 s^−1^ and 2500 s^−1^, indicating that CHM/PDMS composites exhibit pronounced positive strain rate sensitivity.

**Figure 6:**
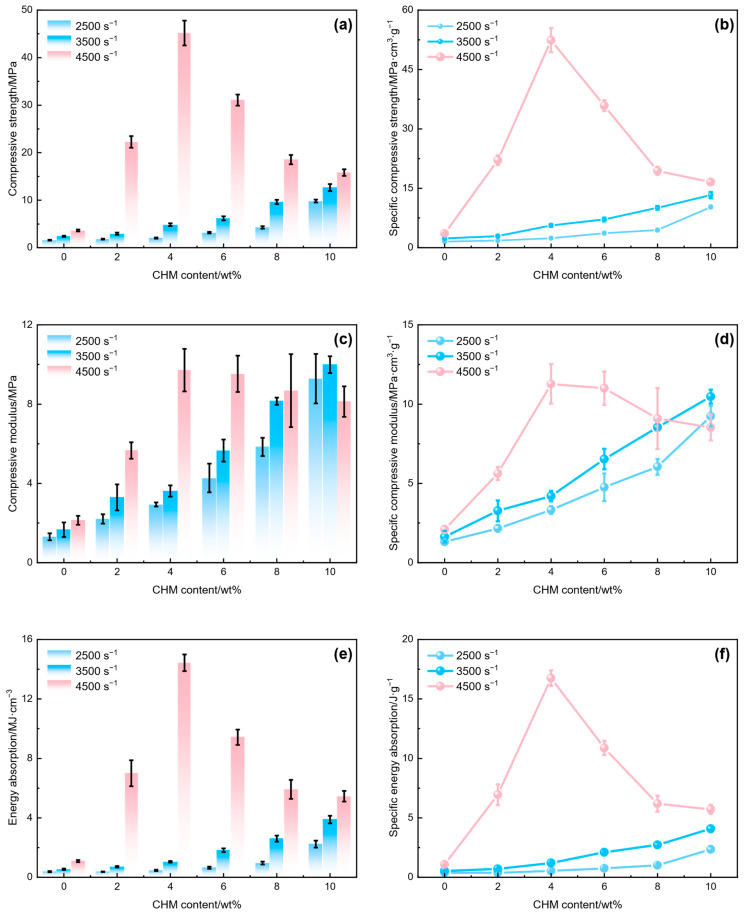
Dynamic impact performance of CHM/PDMS composites: (**a**) compressive modulus; (**b**) compressive strength; (**c**) energy absorption performance; (**d**) specific compressive modulus; (**e**) specific compressive strength; (**f**) energy absorption density.

The impact resistance of CHM/PDMS composites was characterized by their compressive modulus, compressive strength, and energy absorption performance. As shown in Figure 6, increasing CHM content effectively enhances the dynamic mechanical properties of the CHM/PDMS composites at high strain rates. The compressive modulus, compressive strength, and energy absorption values of the CHM/PDMS composites all increase with increasing CHM content. Specifically, Figure 6a,b show that at strain rates of 2500 s^−1^, 3500 s^−1^, and 4500 s^−1^, the compressive modulus, compressive strength, and energy absorption performance of the composites are higher than those of the pure PDMS matrix. Compared with the pure PDMS matrix, when the CHM content is 4 wt.%, the composites exhibit increases in compressive modulus, compressive strength, and energy absorption of 124.68%, 27.70%, and 23.35% at 2500 s^−1^; of 117.44%, 102.76%, and 91.37% at 3500 s^−1^; and of 354.47%, 1245.09%, and 1218.32% at 4500 s^−1^, respectively. The optimal energy absorption performance is assigned to the CHM_04_/PDMS sample at the strain rate of 4500 s^−1^, where the energy absorption value and specific energy absorption value are 14.5 MJ/cm^3^ and 16.7 J/g, respectively. In the PDMS matrix, CHMs act as reinforcement centers that interact with the matrix [30].

As the proportion of the reinforcement centers increases, the strength and stability of the reinforcement network are enhanced [31], which gives rise to a synergistic enhancement of the compressive modulus, compressive strength, and energy absorption performance. Moreover, good dispersion of the CHMs contributed to the enhanced impact resistance. The observations can be explained by the fact that CHMs are dispersed within the PDMS matrix to form energy-absorbing microstructures, which can help to transmit the stress through the interfaces among the CHMs and PDMS, slowing the rate of crack initiation and significantly improving the toughness of the composites. As the CHM loading increases, the crack-bridging effect of the CHMs becomes increasingly pronounced. In summary, the addition of CHMs can improve both the strength and cushioning ability of the PDMS. Increasing the CHM content can lead to enhancements in compressive modulus, compressive strength, and energy absorption at different strain rates, especially for the strain rate of 4500 s^−1^, where the performance improves most notably.

**Figure 7:**
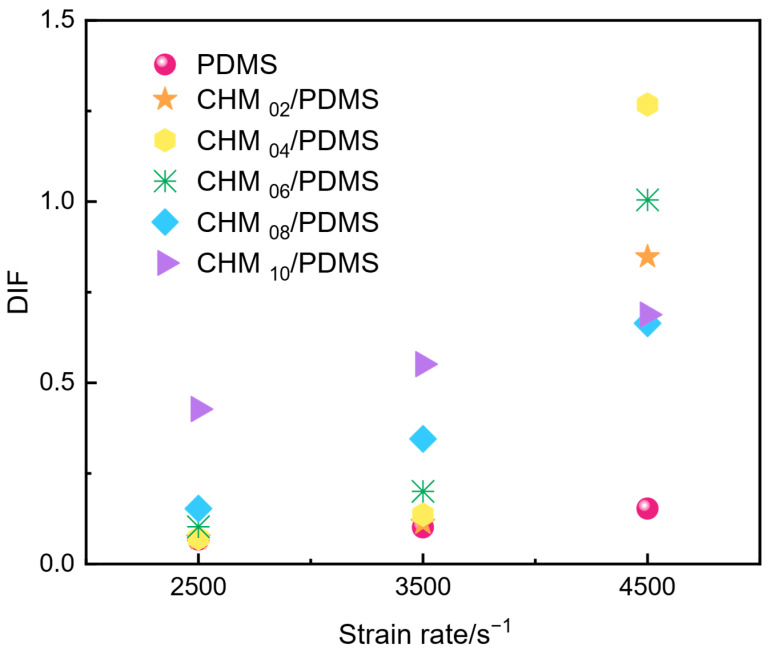
Relationship between dynamic increase factor (DIF) and strain rate for the materials.

The DIF quantifies the enhancement or reduction of the dynamic mechanical properties of the composites compared to their quasi-static properties [32]. As summarized in Figure 7, the DIF values of CHM/PDMS composites with varying filler contents were analyzed at different strain rates. The PDMS matrix, exhibiting inherent strain-rate dependence, shows a pronounced strain rate hardening effect, where DIF values rise sharply with increasing strain rates. Notably, for pure PDMS, the DIF remains nearly constant within the strain rate range of 2500~4500 s^−1^, indicating minimal divergence between its dynamic impact and quasi-static compressive performance. In contrast, the DIF of CHM/PDMS composites increases progressively with CHM content, particularly at enhanced strain rates. For instance, at 4500 s^−1^, the DIF of CHM_04_/PDMS (4 wt.% CHM) and CHM_06_/PDMS (6 wt.% CHM) surpasses that of pure PDMS by 730% and 554%, respectively. This marked enhancement can be attributed to two synergistic mechanisms under high-strain-rate loading: (1) CHMs act as the rigid fillers, absorbing impact energy through elastic deformation of their shell structures, and (2) controlled fracture of microspheres deflects crack propagation paths, delaying the coalescence of primary cracks within the matrix. Additionally, the interaction between CHMs and PDMS caused by the compact stacking effect further improves the impact resistance of the CHM/PDMS composites. However, excessive CHM content (e.g., CHM_10_/PDMS with 10 wt.% filler) results in a reduced strain rate sensitivity. At 3500 s^−1^ and 4500 s^−1^, the DIF values of CHM_10_/PDMS increase by only 29% and 61%, respectively, compared to that at the strain rate of 2500 s^−1^. The lessened strain rate sensitivity could be attributed to filler agglomeration which deteriorates the structural stability of the PDMS matrix, limiting the improvement in the dynamic response.

**Figure 8:**
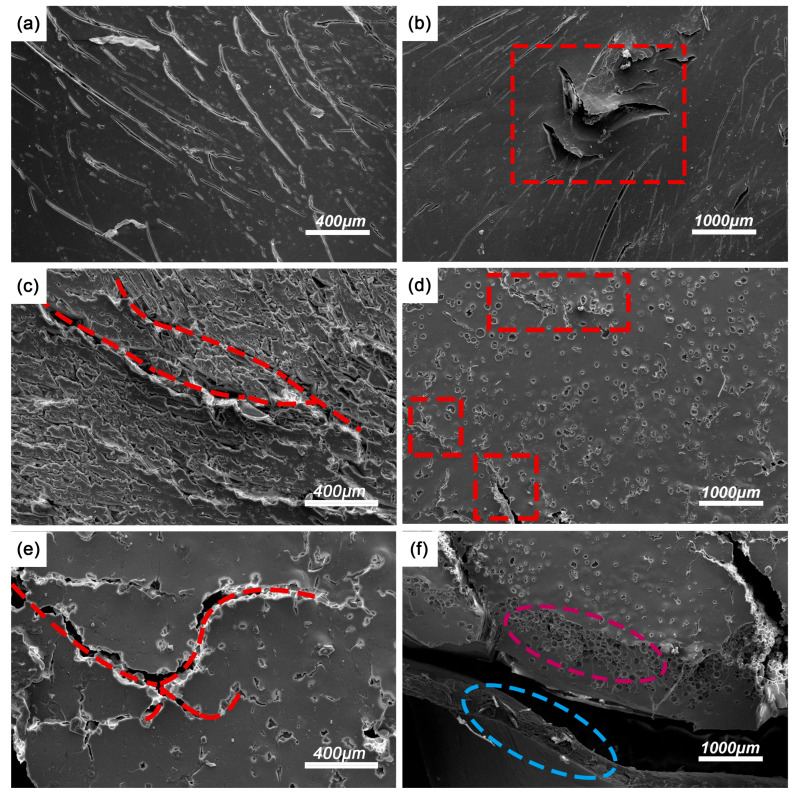
SEM images of the materials under dynamic loading (**a**–**c**): PDMS matrix under impact at strain rates of (**a**) 2500 s^−1^, (**b**) 3500 s^−1^, and (**c**) 4500 s^−1^; (**d**–**f**) CHM_04_/PDMS composite under 2500 s^−1^ strain rate: (**d**) surface morphology, (**e**) crack propagation, and (**f**) fracture surface. The red boxes in the (**b**) and (**d**) represent for the matrix cracks and CHM agglomeration; the red line in the (**c**) and (**e**) represent for the crack propagation path; And the circles in (**f**) displays the matrix deformation (blue circle) and fragmented CHMs (magenta circle).

To further investigate the fracture mechanisms, SEM was utilized to analyze the fracture surfaces of CHM/PDMS composites after dynamic impact loading, as depicted in Figure 8. For the PDMS matrix, at a strain rate of 2500 s^−1^, the fracture surface exhibits a homogeneous morphology with no obvious cracks or pores, indicating the deformation of the pure PDMS under dynamic loading is dominated by the viscoelastic behavior where the energy can be dissipated through the matrix shrinkage or plastic deformation. When the strain rate increases to 3500 s^−1^, the localized strain hardening phenomenon occurs, accompanied by the formation of microcracks (Figure 8b), which could be attributed to the insufficient time for plastic deformation of the polymer matrix. At 4500 s^−1^ (Figure 8c), the fracture surface displays a rough, fibrous texture, signifying the occurrence of shear deformation and crack branching. In other words, with increased strain rate, the deformation mode alters from plastic deformation to localized brittle fracture, verifying the delayed viscoelastic response of PDMS under high strain rates, where the failure mechanisms evolve from ductile to brittle as strain rates increased.

In contrast, the CHM_04_/PDMS composites show distinct fracture characteristics. At 2500 s^−1^, CHMs are relatively well-dispersed with good interfacial interaction through the dense stacking effect. However, with enhanced concentration of the CHMs, localized agglomeration (highlighted in red boxes) could be noticed, acting as stress concentration points (Figure 8d). The crack propagation path, as displayed in Figure 8e, reveals that the fractures can primarily extend along the CHM–PDMS interface, confirming that interfacial interaction was the dominant energy absorption mechanism. Simultaneously, lots of crushed CHMs could be seen along the crack paths, indicating the energy can be dissipated by the deformation of the microspheres. The cross-section morphology of CHM_04_/PDMS (Figure 8f) exhibits a hybrid structure of matrix plastic deformation (blue regions) and fragmented CHMs (magenta regions), suggesting the synergistic energy dissipation via plastic deformation of the matrix and filler crush. Notably, the interfacial gaps can be seen among the microsphere and matrix, indicating that the energy can be dissipated through the interfacial debonding. As the load increases, the densification of crushed CHMs enhances the load-bearing capacity of the composites, underscoring the superior energy dissipation performance of the as-prepared CHM/PDMS composites. In conclusion, the introduction of CHMs can alter the failure mode under dynamic loading: at high strain rates, when the failure mode of the pure PDMS is altered to the brittle fracture, the CHM/PDMS composites exhibit enhanced energy absorption performance through the combination of interfacial debonding, microsphere crushing, and matrix plastic deformation.

**Figure 9:**
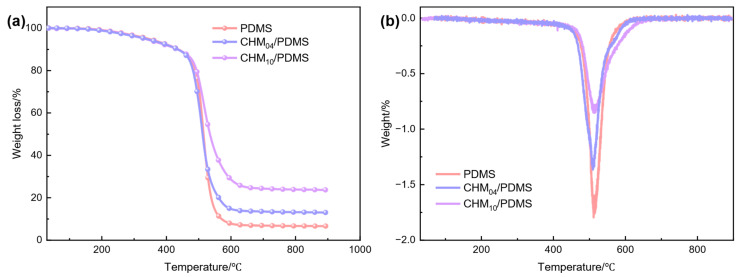
Thermal stability of CHM/PDMS composites: (**a**) TGA curves; (**b**) DTG curves.

TGA analysis was conducted to study the thermal stability of the PDMS and CHM/PDMS composites, as shown in Figure 9. PDMS, CHM_04_/PDMS, and CHM_10_/PDMS exhibit similar thermal degradation trends. The introduction of CHMs significantly increases the onset decomposition temperature of the PDMS matrix. During the thermal decomposition process, three weight loss stages are observed. The first stage occurs in the temperature range of 30~180 °C, followed by the second stage between 180 and 400 °C. The mass loss in the two stages can be attributed to the degradation of the low molecular weight part of the PDMS and other retained impurities, respectively. The final thermal decomposition stage occurs between 460 and 590 °C, during which the weight drops sharply due to the cleavage of Si–CH_3_ bonds. When the temperature exceeds 590 °C, a stable residue is formed. Compared with the pure PDMS matrix, the enhanced remanent weight of the composites can be attributed to the introduction of the CHMs. The DTG curves of the CHM/PDMS composites are shown in Figure 9b. All three curves initially exhibit a flat line in the 0~400 °C range, indicating that there is no significant weight loss in this region. A primary peak can be observed in the range of 460~590 °C, signifying the main decomposition stage of the CHM/PDMS composites, which is constant with the results of the TGA curves. The weight loss during this stage can be attributed to specific depolymerization reactions that result in the elimination of silane and cyclic siloxane units, during which the Si–C, C–C, and C–H bonds break. It may lead to the formation of inorganic residues and the release of siloxane compounds as well as low molecular weight hydrocarbons.

The CHM content has a significant effect on the thermal decomposition temperature *T_d_* and carbon residual rate, which reflect the thermal stability of CHM/PDMS composites. When the CHM content is 10 wt. %, the composite exhibits higher *T_d_* since the CHMs have better thermal stability than the PDMS. The carbon residue rate is also enhanced since CHMs are introduced. The enhancement of *T_d_* and carbon residual rate indicates introducing CHMs to the PDMS matrix gives rise to better thermal stability. When the CHM content is 4 wt. %, the *T_d_* and carbon residual are slightly reduced compared to the sample with high CHM content, but they are still higher with respect to the pure PDMS.

## 4. Conclusions

In this study, CHMs with low breakage rates and high structural stability were fabricated and introduced into the PDMS matrix for enhancing the energy absorption performance. The composite with 4 wt.% CHM exhibits the optimal energy absorption performance, as well as compressive modulus and strength. In samples with low CHM contents (≤4 wt.%), the CHMs were uniformly dispersed within the matrix with strong interfacial interaction caused by the dense stacking effect. However, with higher CHM contents (≥8 wt.%), agglomeration of the CHMs can be found, which induces stress concentration and leads to a deterioration in the energy absorption performance. At a high strain rate of 4500 s^−1^, the CHM/PDMS composite with 4 wt.% CHM shows enhancements in compressive strength and energy absorption values of 1245.09% and 1218.32%, respectively, compared to pure PDMS. The main energy dissipation mechanisms include the elastic deformation of the PDMS, crushing of the CHMs, and their interfacial interaction. In addition, the CHM/PDMS composites exhibit better thermal stability than the pure PDMS, proving that composite materials have the potential for multifunctional applications.

## Data Availability

The original contributions presented in this study are included in the article/Supplementary Material. Further inquiries can be directed to the corresponding author(s).

## Supplementary Materials

The following supporting information can be downloaded at: https://www.mdpi.com/article/10.3390/polym17152087/s1, Figure S1: The SEM images of the phenolic microspheres; Figure S2: The as-prepared CHMs with varied diameter distribution of microspheres.
