# Supplementary material for: Investigation of the Reinforcement Mechanism and Impact Resistance of Carbon Hollow Microsphere-Reinforced PDMS Composites
Source: Polymers (Basel). 2025 Jul 30;17(15):2087. doi: 10.3390/polym17152087 (PMC12349524; doi:10.3390/polym17152087)
Supplement: Supplementary file 1 [file polymers-17-02087-s001.zip › polymers-3773719-supplementary.pdf]

Supplementary information for

Investigation of the Reinforcement Mechanism and Impact Resistance of Carbon Hollow Microsphere Reinforced PDMS Composites

Yingying Yu[a], Yaxi Zhang[a], Cheng Yang[a], Fandong Meng[a,*], Fanyi Meng [a], Tao Wang, Zhenmin Luo[a,*]

[a] College of Safety Science and Engineering, Xi’an University of Science and Technology, Xi’an 710054, China

Figures:

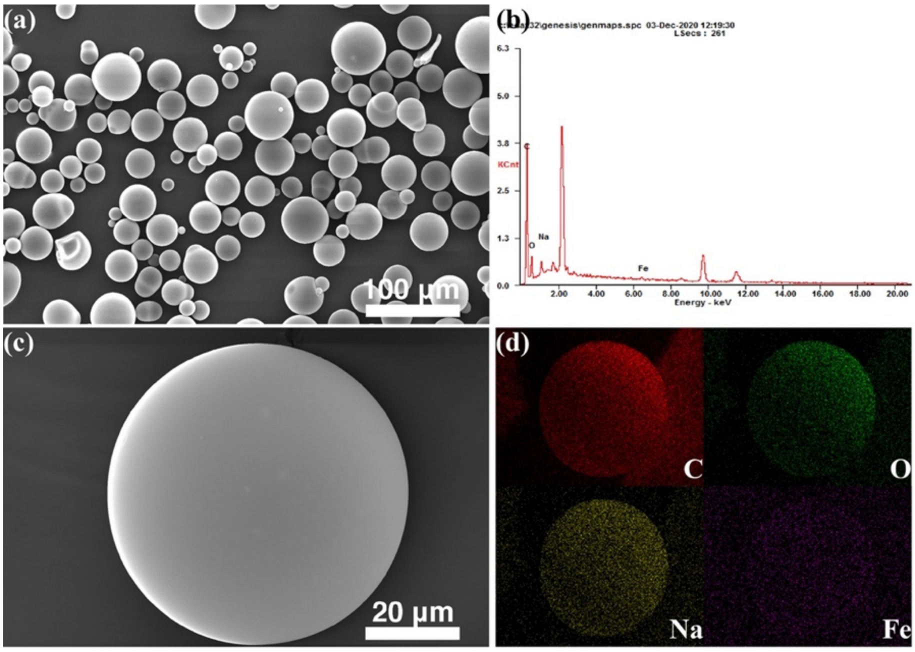

Figure S1 The SEM images of the phenolic microspheres.

* Corresponding author.
Email address: zmluo@xust.edu.cn
* Corresponding author.
Email address: MengF@xust.edu.cn

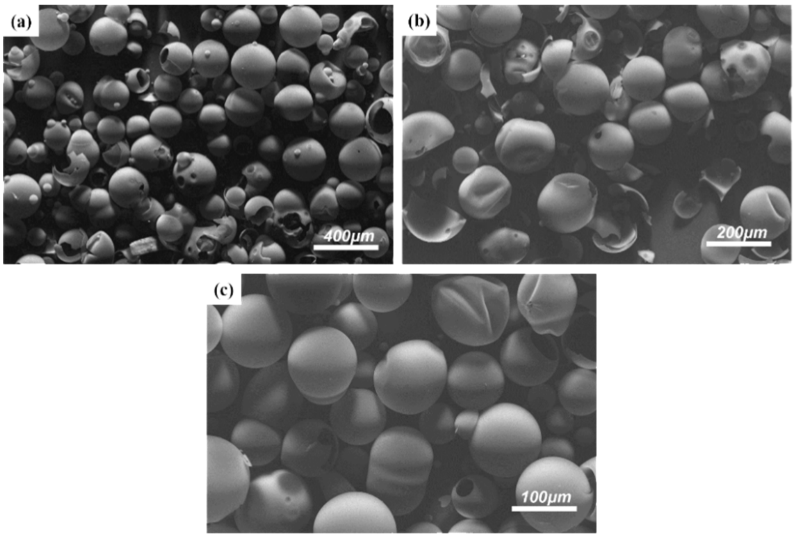

Figure S2 The as-prepared CHMs with varied diameter distribution of microspheres.
